# Impact of Postharvest Oxalic Acid Treatments on Quality Properties and Biochemical Composition of Medlar (*Mespilus germanica* L.) Fruit

**DOI:** 10.1002/fsn3.71362

**Published:** 2026-04-15

**Authors:** Ayşen Melda Çolak, Özlem Gündoğdu, Erdal Aglar, Nevzat Sezgin, Erdal Orman, Muttalip Gundogdu

**Affiliations:** ^1^ Department of Horticulture, Faculty of Agriculture Uşak University Uşak Turkey; ^2^ Republic of Turkey Ministry of Agriculture and Forestry Atatürk Horticultural Central Research Institute Yalova Turkey; ^3^ Department of Landscape Architecture, Faculty of Arts, Design and Architecture Munzur University Tunceli Turkey; ^4^ Department of Horticulture, Faculty of Agriculture Şırnak University Şırnak Turkey; ^5^ Department of Horticulture, Faculty of Agriculture Bolu Abant Izzet Baysal University Bolu Turkey; ^6^ Department of Park and Horiculture, Yalova Vocational School Yalova University Yalova Turkey

**Keywords:** organic acids, phenolic compounds, respiration rate, vitamin C, weight loss

## Abstract

This study was conducted to investigate the effects of oxalic acid (OA) applications at different concentrations on the quality traits and biochemical components of medlar ( *Mespilus germanica* L.) fruit. Fruits harvested in Bolu province in 2024 were subjected to control (0) and 0.5, 1.0, and 1.5 mM OA applications and stored at 4°C and 90% ± 5% relative humidity for 45 days. Samples were taken from the fruits at certain intervals (0, 15, 30, and 45 days) and analyzed. Specifically, 1.0 and 1.5 mM OA applications significantly reduced fruit weight loss and decay rates. OA also inhibited increases in fruit respiration rate and pH and slowed the decline in total acidity, soluble solids content (SSC), vitamin C, phenolic compounds, and organic acids. Malic, citric, succinic, tartaric, and fumaric acids, vitamin C, and important phenolic compounds (chlorogenic acid, gallic acid, quercetin, catechin) were protected by OA applications, and this protection was most pronounced at 1.5 mM OA dose. The correlation analyses revealed significant relationships between quality parameters and biochemical contents. The weight loss and decay rate were negatively correlated with bioactive compounds such as gallic acid and ascorbic acid, indicating that quality deterioration progressed in parallel with the decrease in nutrient content. A strong positive correlation was found between titratable acidity and malic and citric acids. In principal component analysis (PCA), malic and citric acids were prominent in the early storage period, while weight loss, decay, and increased respiration became dominant in the late storage period. Phenolic compounds decreased significantly with storage time, and antioxidant capacity decreased. Heat map analysis visually supported the increase in spoilage parameters with the decrease in ascorbic acid and phenolic compounds. OA applications maintained the quality of medlar fruit and increased biochemical stability, extending postharvest life. 1.5 mM OA, in particular, stands out as an effective postharvest preservation method. These results demonstrate that OA offers significant potential for increasing the commercial value of perishable fruits like medlar and for developing preservation technologies.

## Graphical abstract

Medlar fruits were treated with different concentrations of oxalic acid and stored at 4°C and 90% ± 5% RH for 45 days. In particular, 1.5 mM OA applications effectively reduced weight loss and decay, suppressed increases in respiration rate and pH, and slowed the decline in titratable acidity, SSC. Malic, citric, succinic, tartaric, and fumaric acids, chlorogenic acid, gallic acid, quercetin, catechin, and vitamin C were better preserved with OA applications. Graphical abstract of the effect of oxalic acid application on storage time.

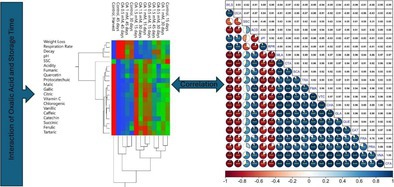

## 1 Introduction

Medlar ( *Mespilus germanica* L.) is a fruit species with high nutritional value that is widely grown in Türkiye, especially in the Western, Northern Anatolia, and Marmara regions (Baytop 1999). Medlar, an important source of health due to its rich antioxidant compounds such as phenolics, flavonoids, ascorbic acid, and tannins, is also used in traditional medicine to treat various ailments. With the increasing health awareness and demand for fresh fruit consumption in recent years, both the nutritional importance and commercial value of medlar are increasing. Medlar, a climacteric fruit species, rapidly undergoes softening, darkening, weight loss, and quality deterioration during the postharvest period (Selcuk and Erkan 2015). This rapid quality loss negatively impacts the fruit's commercial life and consumer acceptance. For this reason, the studies to extend the postharvest life of the Medlar are of great importance.

Oxalic acid is an organic acid found naturally in many plants and stands out for its potential to protect fruit quality, particularly in postharvest applications. The studies have shown that OA exhibits antioxidant properties, delays fruit ripening, prevents cell wall destruction, and preserves fruit appearance by suppressing enzymatic browning (Wu et al. 2011). It has been reported that it delays softening in fruits such as plum, mango, and peach by reducing the activity of softening enzymes, especially polygalacturonase (PG) and pectin methyl esterase (PE) (Zheng et al. 2012). In addition, it is stated that OA application limits the damage of reactive oxygen species (ROS) and protects cellular integrity by increasing the activity of antioxidant defense enzymes such as superoxide dismutase (SOD), peroxidase (POD), and catalase (CAT) (Huang et al. 2013). It is also reported in the literature that OA reduces respiration rate by suppressing ethylene production, thus extending postharvest life (Wang et al. 2022). Another important effect of OA in the postharvest period is its inhibition of microbial spoilage in the fruit. As the systemic resistance‐enhancing agent, OA strengthens fruit resistance against pathogens by increasing the activity of defense enzymes such as phenylalanine ammonia lyase (PAL), polyphenol oxidase (PPO), and POD (Zhu et al. 2016). Thus, OA applications not only positively affect the quality parameters but also support the functional properties of the fruit by maintaining the stability of biochemical components such as ascorbic acid, phenolic compounds, and organic acids (Martínez‐Espla et al. 2014; Ahmed et al. 2021). It is thunk that OA applications can improve marketability by minimizing quality losses, particularly in fruits like medlar, which have limited fresh consumption but high bioactive compound content. Based on this idea, the aim of our study was to investigate the effects of postharvest oxalic acid application on fruit quality traits and biochemical compounds in medlar fruit.

## 2 Material and Methods

### 2.1 Plant Material

Medlar ( *M. germanica* L.) fruits used in this study were harvested from 12‐year‐old trees in Bolu province in 2024. Trees were planted at 5 × 5 m spacing and distance, and cultural practices such as annual pruning, irrigation, fertilization, soil cultivation, and disease and pest control were carried out regularly. Fruits that had reached harvest maturity (identified by the change of peel color from greenish‐brown to light brown, hard seed color turning brown, and firmness of approximately 45–55 N) and showed no physical damage or disease symptoms were hand‐harvested and transported to the Horticulture Laboratory of Bolu İzzet Baysal University's Faculty of Agriculture within 20 min using a refrigerated vehicle (4°C) to prevent temperature fluctuations. A total of 720 fruits were used in the experiment, with three replicates for each treatment and 20 fruits per replicate.

### 2.2 Treatments

Harvested medlar fruits were first carefully washed with distilled water to remove surface dust, dirt, and microorganisms. They were then left at room temperature until the surface was dry. The cleaned fruits were then divided into four different processing groups and subjected to oxalic acid treatments. The treatment groups were determined such that, in the control group, fruits were immersed in distilled water for only 10 min, while the other groups were immersed in OA solutions at concentrations of 0.5, 1.0, and 1.5 mM, respectively, for 10 min. The ratio between fruit and OA solution was 1:10 (w/v) to ensure complete immersion and uniform exposure. Throughout the treatment period, the temperature of the solutions was kept constant at ambient conditions, and all treatments were carried out under the same conditions. After dipping, the fruits were air‐dried at room temperature to remove excess moisture and ensure surface dryness. The completely dried fruits were placed in polyethylene bags and stored in cold storage. The polyethylene bags used for packaging were 30 μm thick, with water vapor permeability of 10^−11^ g m^−2^ s^−1^ Pa^−1^ and O_2_ permeability of 2500 cm^3^ m^−2^ day^−1^ atm^−1^. The storage was carried out for 45 days at 4°C and 90% ± 5% relative humidity. To monitor fruit quality characteristics throughout the trial, analyses were conducted at four different times: harvest day (Day 0), 15th, 30th, and 45th days.

### 2.3 Weight Loss

Weight loss was determined using 20 fruits per replicate (*n* = 3) by weighing before and after each storage interval. The weights for each fruit sample were measured before storage (initial) and at the end of the analysis day (15th, 30th, and 45th day) using a precision balance (±0.01 g accuracy) and calculated as a percentage using the following formula:

$$\begin{array}{c} \text{Weight loss}\;\left(\%\right)=\left[\left(\text{Initial weight}–\text{Final weight}\right)/\text{Initial weight}\right]\\ \;\times 100 \end{array}$$

### 2.4 Decay Rate

At each analysis period, 30 fruits from each group were randomly selected and subjected to visual evaluation. The decay rate was determined as the ratio of fruits showing visible signs of decay, softening, mold, or staining on the surface or internal tissue due to fungal, bacterial, or physiological deterioration to the total number of fruits. Decay severity was scored using a 0–4 scale (0 = no decay, 1 = slight [1%–10%], 2 = moderate [11%–25%], 3 = severe [26%–50%], 4 = extensive [> 50%] decay). Results were expressed as percentages (%).

### 2.5 SSC, Titratable Acidity, pH, and Vitamin C

For SSC, pH, titratable acidity and ascorbic acid analyses, a total of 90 fruits were selected from each replicate group and divided into three subgroups of 30. Fruits were blended without peeling to represent total edible tissue. After removing the seeds of each fruit, juice was obtained using an electric juicer (HR1855/70, Philips, Türkiye). SSC measurement was performed with a digital refractometer (PAL‐1, McCormick Fruit Tech., Yakima, USA) and expressed as a percentage. pH value was measured with a digital pH meter (Metler Toledo). For acidity determination, 10 mL of fruit juice sample was diluted with an equal volume of pure water and titrated with 0.1 mol/L NaOH solution until pH 8.2 was reached and the results were calculated as %. For the determination of vitamin C, a sufficient amount of fruit juice sample was taken and the total volume was completed to 5 mL with 0.5% oxalic acid. The ascorbic acid test strip from Merck (Catalog No: 116981, Germany) was immersed in the solution for 2 s, waited for 8 s, and then measured with the Merck RQflex plus 10 reflectometer. The results were expressed on a fresh weight (FW) basis (mg 100 g^−1^ FW).

### 2.6 Respiration Rate

The respiration rate was determined at 4°C using 2 L airtight containers equipped with a gas sampling port. Ten fruits were placed in airtight containers and after 2 h of waiting, the amount of CO_2_ released into the environment was measured with the Headspace Gas Analyzer GS3/L (Gasboard‐3210Plus). Respiration rate is expressed in nmol CO_2_ kg^−1^ h^−1^h units.

$$\text{Respiration rate}=\left(V_{j}–V_{f}\right)\times \%{\mathrm{CO}}_{2}\times 10/\mathrm{FW}\times T$$

where *V*_j_: jar volume, *V*_f_: fruit volume, FW: fruit weight (kg), *T*: time (h).

### 2.7 Organic Acids

15 g of fruit sample was mixed with 0.009 N H_2_SO_4_ at a ratio of 1:1 to make it homogeneous and kept on a shaker for 1 h. The resulting mixture was centrifuged at 15,000 rpm for 15 min, and the upper solution was collected. This solution was made suitable for HPLC analysis by passing through a 0.45 μm membrane filter and a SEP‐PAK C18 cartridge, respectively. Organic acid analysis was performed using an Agilent HPLC 1100 series G 1322 A (Germany) instrument with an Aminex HPX‐87H column (300 mm × 7.8 mm, Bio‐Rad Laboratories, USA) according to the method recommended by Bevilacqua and Califano (1989). Analyses were conducted using fresh fruit pulp samples, and results were calculated on a fresh weight basis, and the results are expressed as g kg^−1^, but for fumaric acid as mg 100 g^−1^.

### 2.8 Individual Phenolic Compounds

The analysis of phenolic compounds at the individual level was carried out according to the method developed by Rodrıguez‐Delgado et al. (2001). Five grams of medlar pulp were homogenized with an equal volume of distilled water at 4°C before centrifugation and centrifuged at 15,000 rpm for 15 min. The obtained supernatant was first filtered through a coarse filter, then through a 0.45 μm membrane filter and injected into the HPLC device (Agilent HPLC 1100 series G 1322 A, Germany). The separation process was carried out with an ODS column (HiChrom, USA) with a DAD detector and a size of 250 × 4.6 mm, containing 4 μm particles. The results were expressed on a fresh weight (FW) basis (mg kg^−1^. 100 g^−1^ FW).

### 2.9 Statistical Analysis

The data obtained from the study were evaluated statistically using variance analysis (ANOVA). The differences between applications were considered significant at *p* < 0.05 and compared using the Tukey test. All analyses were performed using SAS Version 9.1 (SAS Institute Inc., Cary, NC, USA) statistical software. The correlation analysis and principal component analysis (PCA) were performed using Jmp Pro 17 software.

## 3 Results and Discussion

### 3.1 Weight Loss

**FIGURE 1:**
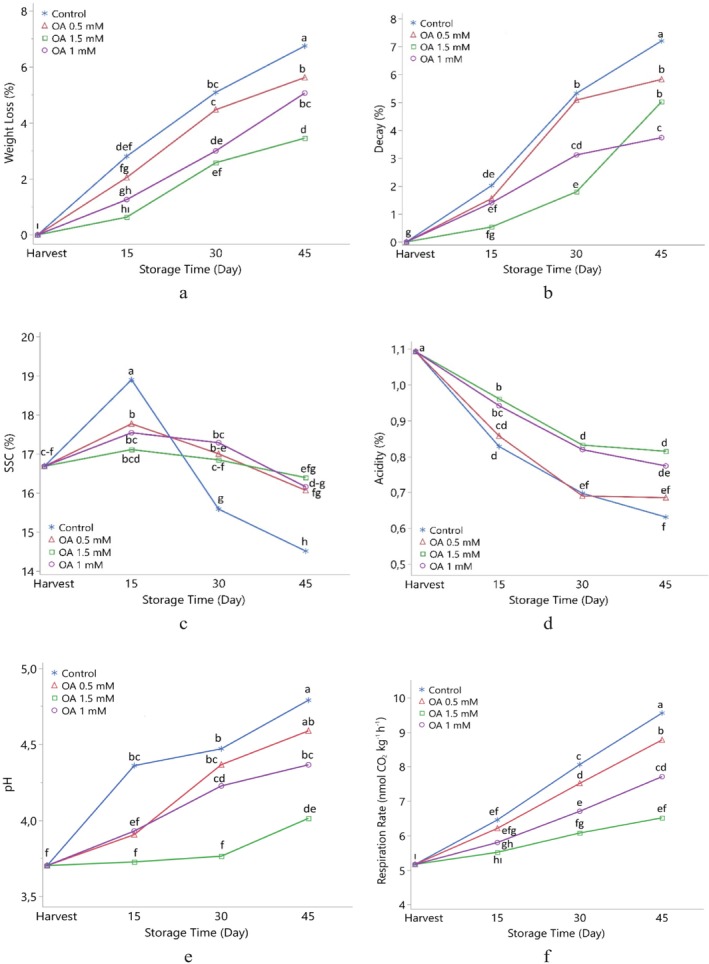
Effect of oxalic acid application on weight loss (a), decay (b), SSC (c), acidity (d), pH (e), and respiration rate (f) of medlar fruit during storage. Different letters on top of the storage periods indicate significant differences at *p* ≤ 0.05. SSC, solouble solid content.

The weight loss in medlar fruits increased significantly as storage progressed. This loss, which was 1.68% on the 15th day of storage, reached 3.78% on Day 30 and 5.21% on Day 45, indicating that the fruits lose mass over time due to both physiological water loss and continued metabolic activity. This increase can be explained by water loss, generally through transpiration from the fruit surface, along with cell structure deterioration and continued respiratory activity due to ripening and aging. However, OA applications have been found to significantly reduce the weight loss. In particular, the minimal loss of only 0.63% was detected at the end of Day 15 with 1.5 mM OA application, compared to the 2.80% loss in the control group, demonstrating an effective protective function (Table S1 and Figure 1). These results may be related to the effects of OA in slowing water loss from the fruit surface, reducing cell membrane permeability, and suppressing metabolic activities. OA can delay fruit ripening and senescence by increasing antioxidant enzyme activities (Zheng et al. 2007) and decreasing ethylene production (Wang et al. 2009). These mechanisms contribute to water conservation in internal fruit tissues and maintenance of cell membrane integrity. The basis for this protective effect of OA is its ability to reduce oxidative damage and maintain cellular integrity by suppressing reactive oxygen species (ROS) accumulated in the fruit (Razzaq et al. 2014). It is also thought that OA delays aging‐related cell destruction by increasing the activities of antioxidant enzymes such as SOD, CAT, and peroxidase (POX), thus maintaining a more metabolically stable structure and limited water loss in the fruit (Huang et al. 2013). This effect has been reported similarly in many fruits such as mango, plum, longan, and peach (Zheng et al. 2012; Wu et al. 2011; Khan et al. 2020). Similar findings revealed that the strength of cell walls increased and water loss through evaporation decreased with hardening of pericarp tissues in OA‐treated fruits (Zhu et al. 2016). It is stated that preharvest OA applications similarly slow down the metabolic processes of the fruit, increase calcium accumulation, provide cell membrane stability, and thus delay both weight loss and quality loss (Martínez‐Espla et al. 2014; García‐Pastor et al. 2021). In addition, it is reported that OA suppresses fruit senescence by creating systemic resistance, providing redox homeostasis and regulating ethylene signaling pathways (Wang et al. 2012; Li et al. 2022).

### 3.2 Decay Rate

The rate of decay in medlar fruits increases significantly and steadily with extended storage. The rate of decay, which was 0% at harvest, increased to 1.38% on the 15th day, 3.83% on the 30th day, and 5.44% on the 45th day of the cold storage. This increase suggests that physiological deterioration and microbial activity in fruit tissue accelerate over time. These results can be explained by the weakening of the fruit's structural defenses during storage and its increased susceptibility to microbial infection due to environmental conditions. Humid and low‐temperature environments, in particular, trigger fungal growth on the stem and peel surfaces, increasing the rate of decay. Furthermore, the loss of cell membrane integrity during storage facilitates the penetration of pathogens into the fruit and accelerates spoilage. In this context, OA applications significantly reduced decay rates, particularly at concentrations of 1.0 and 1.5 mM. For example, on the 15th day, the decay rate was only 0.53% in the OA 1.5 mM group, while this rate reached 2.02% in the control group. Similarly, the low decay rates in the OA‐treated groups remained on subsequent days, demonstrating the continued protective effect of OA (Table 1 and Figure 1). This effect of OA is due to its capacity to activate defense systems in the fruit. The literature has shown that OA increases tissue strength by reducing the activity of cell wall‐degrading enzymes such as PG and PE and creates a physiological barrier against microorganisms (Wu et al. 2011). It is also known that OA inhibits the accumulation of reactive oxygen species by increasing the activity of antioxidant enzymes such as superoxide dismutase, peroxidase, and catalase (Ding et al. 2007). The effect of OA application in suppressing decay is not only limited to preserving cell structure but also directly inhibits pathogen development. OA, as an abiotic stimulus, is reported to trigger systemic resistance mechanisms (Mucharromah and Kuc 1991), increasing the production of defense‐related enzymes such as PAL, β‐1,3‐glucanase, and chitinase. For example, in a study on kiwifruit, OA application reduced blue mold ( *P. expansum* ) development and patulin accumulation, and significantly reduced the rate of rotten tissue (Martínez‐Espla et al. 2014).

**TABLE 1:** Impact of postharvest oxalic acid application on organic acids and vitamin C contents of medlar fruits (mg 100 g^−1^).

Storage time		Malic	Citric	Succinic	Tartaric	Fumaric	Vitamin C
Harvest		650.56 ± 7.30a	486.26 ± 12.19a	164.66 ± 3.17a	97.10 ± 2.55a	5.46 ± 0.15a	76.29 ± 1.98a
Day 15		584.72 ± 6.79b	410.52 ± 13.02b	148.53 ± 2.29b	85.71 ± 2.68a	3.82 ± 0.24b	64.42 ± 1.34b
Day 30		538.32 ± 9.09c	329.48 ± 11.39c	142.42 ± 2.16b	70.00 ± 4.53b	2.75 ± 0.19c	56.75 ± 1.11c
Day 45		467.51 ± 7.62d	277.61 ± 14.69d	125.18 ± 2.75c	62.09 ± 4.49b	2.39 ± 0.19c	51.19 ± 1.30d
Oxalic acid and storage time interaction							
Harvest		650.56 ± 7.30a	486.26 ± 12.19a	164.66 ± 3.17a	97.10 ± 2.55a	5.46 ± 0.15a	76.29 ± 1.98a
Day 15	Control	561.31 ± 8.61ef	363.91 ± 8.13d	142.32 ± 1.71de	75.67 ± 1.19de	2.86 ± 0.10d	59.14 ± 1.54de
	OA 0.5 mM	582.28 ± 5.18cd	394.61 ± 6.32c	144.60 ± 2.82cd	83.99 ± 1.80bc	3.67 ± 0.13c	63.92 ± 1.16c
	OA 1.0 mM	588.03 ± 6.66c	432.53 ± 0.90b	150.63 ± 2.24bc	88.54 ± 0.43b	4.23 ± 0.15b	66.72 ± 0.67bc
	OA1.5 mM	607.26 ± 8.48b	451.04 ± 9.48b	156.57 ± 2.89b	94.64 ± 2.19a	4.52 ± 0.15b	67.91 ± 0.52b
Day 30	Control	503.05 ± 6.81h	295.73 ± 7.68g	134.01 ± 2.88fg	50.53 ± 2.46g	2.13 ± 0.08f	52.68 ± 0.44gh
	OA 0.5 mM	535.74 ± 4.67g	308.37 ± 3.16fg	141.93 ± 1.91de	70.72 ± 1.11e	2.50 ± 0.09e	56.18 ± 0.83ef
	OA 1.0 mM	546.64 ± 4.61fg	342.23 ± 4.51e	146.66 ± 1.71cd	78.36 ± 1.97cd	2.91 ± 0.06d	57.69 ± 0.32def
	OA1.5 mM	567.86 ± 5.46de	371.58 ± 4.97d	147.10 ± 2.32cd	80.38 ± 2.56cd	3.46 ± 0.06c	60.44 ± 1.37d
Day 45	Control	438.89 ± 6.33j	220.56 ± 7.45ı	117.74 ± 2.97ı	46.86 ± 3.29g	1.70 ± 0.09g	47.00 ± 1.19ı
	OA 0.5 mM	465.49 ± 4.01ı	265.48 ± 3.61h	121.05 ± 2.98hı	58.88 ± 0.32f	2.25 ± 0.06ef	49.41 ± 0.97hı
	OA 1.0 mM	472.44 ± 4.70ı	305.21 ± 5.42fg	126.32 ± 1.98gh	62.99 ± 1.26f	2.54 ± 0.08e	52.71 ± 0.78gh
	OA1.5 mM	493.22 ± 6.22h	319.21 ± 6.77f	135.61 ± 2.72ef	79.64 ± 1.03cd	3.06 ± 0.07d	55.65 ± 1.30fg
ANOVA							
*F* (Storage time)		57.28***	28.11***	26.33***	9.31***	20.14***	36.78***
*F* (OA × Storage time)		98.38***	125.44***	28.68***	68.66***	104.65***	54.65***

*Note:* Different letters in the same column indicates statistical differences at *p* ≤ 0.05.

Abbreviation: OA, oxalic acid.

*** *p* ≤ 0.001.

### 3.3 SSC, Titratable Acidity, and pH

The SSC of medlar fruit, determined as 16.68% at harvest, increased to 17.82% on the 15th day of storage and then decreased to 15.78% on the 30th and 45th days. This increase can initially be attributed to the formation of free sugars through starch breakdown while the decrease can be attributed to the consumption of sugars during respiration and metabolic processes. OA applications limited this downward trend; particularly, 0.5 and 1.0 mM concentrations maintained higher SSC levels compared to the control group (Table S1 and Figure 1). This effect may be attributed to the properties of OA in delaying fruit ripening and protecting cell wall integrity (Wu et al. 2011). It has been shown that OA reduces sugar losses by suppressing PG and PE enzyme activities, especially in mango (Zheng et al. 2012) and plum (Wu et al. 2011). Thus, OA application stands out as an important preservation strategy that reduces quality loss by balancing sugar metabolism. Total acidity values were recorded at 1.09% at harvest and 0.73% at the end of the 45th day of storage. This decrease is related to the use of organic acids as substrates during respiration. OA, particularly at 1.0 and 1.5 mM doses, slowed this decline. It is thought that OA limits acid consumption by suppressing respiration and preserves acids for longer periods by regulating cellular metabolism. Ding et al. (2007) and Tian et al. (2006) reported that OA application contributes to the preservation of organic acids by slowing down oxidative processes within the fruit. Furthermore, acid balance was reported to be more stable throughout the shelf life of artichokes and pears with OA application (Ruíz‐Jim'enez et al. 2014; Tian et al. 2006).

The pH, which was initially 3.70, increased to 4.44 on the 45th of storage. This increase is directly proportional to the decrease in acidity and indicates the depletion of organic acids in the fruit. OA applications slowed down this increase in pH. 1.5 mM OA, in particular, kept the pH lower (Table 1 and Figure 1). OA's inherent acidic structure, antimetabolic effects, and antioxidant properties that suppress ROS production explain this result (Huang et al. 2013). It has been reported that after OA application in mango and peach, the pH increase is limited and the acidic balance within the fruit is maintained (Wu et al. 2011). The findings of this study indicate that OA contributes to the preservation of quality parameters in medlar fruit by delaying both physiological ripening and biochemical deterioration. The literature has reported that OA has multiple effects, including suppressing softening enzymes such as PG and PE, activating antioxidant enzymes, and reducing sugar and acid loss by limiting respiration (Zheng et al. 2012; Martínez‐Espla et al. 2014).

### 3.4 Respiration Rate

It was determined that the respiration rate of medlar fruit increased significantly during storage. The respiration rate, initially measured as 5.17 nmol CO_2_ kg^−1^ h^−1^, reached 8.14 nmol CO_2_ kg^−1^ h^−1^ on the 45th day of storage (Table S1 and Figure 1). This increase is a typical indicator of accelerated metabolic activity during ripening and aging. Fruit respiration is directly linked to enzymatic and oxidative processes that accompany ethylene production, and these processes trigger quality deterioration such as fruit softening, nutrient loss, and decay. This increased respiration in medlar fruit may have been due to increased reactive oxygen species production during ripening, ethylene signaling, and activation of enzymes that mediate cell wall degradation. Many studies in the literature have reported that such increases are associated with cellular redox imbalance and membrane structure disruption, and that oxidative stress in fruit tissues increases (Razzaq et al. 2013). In this context, OA applications showed a significant suppressive effect on fruit respiration. Particularly, 1.5 mM OA concentration contributed to the slowing of metabolic activity in fruit by reducing the respiration rate to the level of approximately 6.00 nmol CO_2_ kg^−1^ h^−1^ (Table S1 and Figure 1). This effect of OA is explained by mechanisms such as increasing antioxidant enzyme activities, ROS detoxification and preserving cell membrane stability, which are frequently emphasized in the literature (Huang et al. 2013). OA also delays fruit ripening by suppressing ethylene biosynthesis (Li et al. 2022), which indirectly reduces respiration rate. These findings are consistent with previous studies on fruits such as mango (Zheng et al. 2007), plum (Wu et al. 2011), peach (Zheng and Tian 2006), and kiwi (Ahmed et al. 2021). For example, Martínez‐Espla et al. (2014) showed that OA applications in plum fruit suppressed both fruit softening and respiration rate by reducing PG and PE activities. Similarly, Serna‐Escolano et al. (2021) reported that OA suppressed respiration peaks and stabilized fruit metabolism in artichoke.

### 3.5 Organic Acids

It was observed that the amounts of malic, citric, succinic, tartaric, and fumaric acids, as well as ascorbic acid (vitamin C), determined in medlar fruit decreased during storage. The highest amount of malic acid (650.56 mg 100 g^−1^) was determined, followed by citric, succinic, tartaric, and fumaric acids, respectively. The decreases in malic, citric, and succinic acids were particularly pronounced while fumaric acid was detected at the lowest levels and showed a dramatic decrease throughout storage. Ascorbic acid content also decreased from 76.29 to 51.19 mg 100 g^−1^ at the end of Day 45. These decreases were associated with postharvest physiological processes such as ripening, increased respiration, and oxidative degradation. In this context, it was observed that OA applications, especially at concentrations of 1.0 and 1.5 mM, slowed down the losses in the compounds in question and preserved biochemical stability (Table 1). The slowing of the decline in malic acid by OA suggests that OA may inhibit the metabolism of this compound, which is used in the TCA cycle for energy production. Similarly, the preservation of citric acid can be explained by OA's effect on cellular pH balance and regulation of enzymatic activity. The preservation of even low‐level compounds such as fumaric acid by OA applications demonstrates that OA can stabilize not only major acids but also biologically important minor compounds. In addition, the reduction of ascorbic acid losses by OA has been associated with the activating effect of this organic acid on antioxidant defense systems.

These results are also supported by the studies conducted different fruit species in the literature. For example, as reported by Zheng et al. (2012), it has been releaved that OA reduce the ripening rate by suppressing the activity of cell wall enzymes such as PG and PE in fruits such as mango and plum, thus preserving fruit firmness and component stability. Similarly, Ding et al. (2007) reported that OA limits oxidative degradation by increasing antioxidant enzyme activities. These effects of OA have been associated with limiting ROS accumulation, increasing the stability of cell membranes and maintaining redox balance (Razzaq et al. 2014). In particular, OA applications combined with controlled atmosphere have been shown to suppress phenolic oxidation, browning and pathogen development, and this effect occurs through defense enzymes such as phenylalanine ammonia lyase, peroxidase and polyphenol oxidase (Ali et al. 2020; Khan et al. 2020).

### 3.6 Specific Phenolic Compounds

During the 45‐day postharvest storage period, significant decreases were observed in all phenolic compounds such as chlorogenic acid, gallic acid, quercetin, catechin, ferulic acid, protocatechuic acid, vanillic acid, and caffeic acid (*p* ≤ 0.001) (Tables 2 and 3). These decreases observed in phenolic compounds during storage are associated with the natural oxidative stress occurring in fruit tissues, enzymatic degradation, and the activity of enzymes such as phenolic oxidase (PPO) and peroxidase (POD). These enzymes degrade phenolic compounds through oxidative reactions, causing browning, textural deterioration, and nutrient loss in fruits (Mondal et al. 2004; Zheng and Tian 2006). Furthermore, reactive oxygen species (ROS) disrupt the structural integrity of these compounds, leading to a decrease in phenolic content (Razzaq et al. 2014). Phenolic structures become susceptible to oxidative stress, particularly with the accumulation of ROS during storage. However, OA applications significantly slowed this reduction, with the high dose of 1.5 mM OA appearing to be the most effective treatment for preserving phenolic compounds. When evaluated in terms of chlorogenic acid, the value of the fruit, which was 76.07 mg kg^−1^ at harvest time, decreased to 36.87 mg kg^−1^ at the end of Day 45. However, with the application of 1.5 mM OA, the level of this compound was maintained at a very high level of 64.53 mg kg^−1^ on the 15th day of the storage. The interaction between OA and storage time was statistically highly significant (*F* = 45.26), demonstrating that OA significantly suppressed oxidative losses. Gallic acid levels similarly decreased over time. The value, which was 12.99 mg kg^−1^ at harvest, decreased to 8.75 mg kg^−1^ on the 45th day of the storage. However, thanks to 1.5 mM OA application, it remained at 12.02 mg kg^−1^ on the 15th day of the storage. It is understood that OA maintains the structural stability of gallic acid, thus ensuring the sustainability of the phenolic content. This interaction was also found to be highly significant (*F* = 138.4). Quercetin levels also decreased gradually over time, from 6.42 mg kg^−1^ at harvest to 5.10 mg kg^−1^ by the end of Day 45. However, OA treatments, particularly the 1.5 mM dose, maintained quercetin levels at higher levels. The interaction between OA and time was significant (*F* = 55.4), indicating that OA played an active role in preserving the phenolic structure. Catechin decreased from 3.82 to 2.60 mg kg^−1^ during storage, but OA applications also reduced this loss. With 1.5 mM OA application, catechin level was maintained at a higher value of 3.54 mg kg^−1^ on the 15th of storage. The interaction between OA and storage time was highly significant (*F* = 304.95) and the protective effect of OA on catechin was quite evident. The amount of ferulic acid also decreased over time. The value, which was 5.15 mg kg^−1^ at harvest time, decreased to 3.92 mg kg^−1^ at the end of the 45th day. 1.5 mM OA application significantly limited this decrease to 4.93 mg kg^−1^ on the 15th day of the storage. The protective effect of OA application on ferulic acid was found to be significant (*F* = 83.48). Protocatechuic acid also tends to decrease during storage. Although the level, which was 10.62 mg kg^−1^ at harvest, decreased to 5.05 mg kg^−1^ on the 45th day of the storage, 1.5 mM OA application maintained this value up to 8.80 mg kg^−1^ on the 15th day of the storage. The interaction between OA application and time was highly significant (*F* = 124.87), clearly demonstrating the stabilizing effect of OA on the phenolic structure. The similar decreasing trend was observed for vanillic acid. The amount, which was 8.05 mg kg^−1^ at harvest, decreased to 3.98 mg kg^−1^ on the 45th day while 1.5 mM OA application kept this value at 6.92 mg kg^−1^ on the 15th day of the storage. The interaction between OA and time was also found to be significant for this compound (*F* = 81.94). Caffeic acid was one of the compounds showing the sharpest decrease in the study. The decrease occurred from 3.73 to 1.86 mg kg^−1^. However, OA applications slowed this loss, and 1.5 mM OA maintained the loss at 3.22 mg kg^−1^ on the 15th day of the storage. This interaction was also highly significant (*F* = 85.82). Generally, there is the natural tendency for all phenolic compounds in medlar fruit to decrease during storage. However, oxalic acid treatments significantly slowed this decrease, allowing the phenolic compounds to remain more resistant to oxidative and enzymatic degradation. It was determined that 1.5 mM OA dose provided the most effective results for all phenolic compounds. The very high level of statistical significance (*p* ≤ 0.001) in all OA × time interactions suggests that oxalic acid is an effective postharvest protection strategy in preventing the loss of phenolic compounds (Tables 2 and 3). The effectiveness of OA in slowing down this phenolic loss can be explained by the following mechanisms: Anti‐darkening effect: OA helps preserve these compounds by suppressing the activity of enzymes such as PPO and POD, which prevent the oxidative destruction of phenolic compounds (Lin et al. 2017).

**TABLE 2:** Impact of postharvest oxalic acid application on phenolic compounds of medlar fruits (mg kg^−1^).

Storage time		Chlorogenic	Gallic	Quercetin	Catechin
Harvest		76.07 ± 3.72a	12.99 ± 0.25a	6.42 ± 0.11a	3.82 ± 0.03a
Day 15		59.36 ± 1.38b	11.34 ± 0.25b	5.69 ± 0.07b	3.41 ± 0.04b
Day 30		46.94 ± 2.74c	10.14 ± 0.20c	5.30 ± 0.09c	2.87 ± 0.06c
Day 45		36.87 ± 2.25d	8.75 ± 0.13d	5.10 ± 0.14c	2.60 ± 0.04d
Oxalic acid and storage time interaction					
Harvest		76.07 ± 3.72a	12.99 ± 0.25a	6.42 ± 0.11a	3.82 ± 0.03a
Day 15	Control	55.26 ± 2.12cd	10.27 ± 0.15ef	5.54 ± 0.04cde	3.25 ± 0.04d
	OA 0.5 mM	58.98 ± 1.28bc	11.42 ± 0.11c	5.58 ± 0.03cd	3.39 ± 0.03c
	OA 1.0 mM	58.66 ± 0.96bc	11.66 ± 0.11bc	5.68 ± 0.06c	3.45 ± 0.02c
	OA1.5 mM	64.53 ± 1.54b	12.02 ± 0.09b	5.96 ± 0.09b	3.54 ± 0.01b
Day 30	Control	35.00 ± 1.54hı	9.46 ± 0.06g	5.02 ± 0.07f	2.64 ± 0.02g
	OA 0.5 mM	48.33 ± 1.36ef	9.92 ± 0.09f	5.14 ± 0.05f	2.79 ± 0.02f
	OA 1.0 mM	51.05 ± 1.20de	10.34 ± 0.13e	5.45 ± 0.05de	3.01 ± 0.03e
	OA1.5 mM	53.37 ± 1.12cde	10.83 ± 0.04d	5.59 ± 0.01cd	3.04 ± 0.02e
Day 45	Control	29.89 ± 1.52ı	8.26 ± 0.10ı	4.57 ± 0.08g	2.46 ± 0.01ı
	OA 0.5 mM	33.90 ± 0.88hı	8.69 ± 0.08h	4.97 ± 0.03f	2.55 ± 0.01h
	OA 1.0 mM	39.17 ± 1.38gh	9.03 ± 0.08h	5.37 ± 0.03e	2.62 ± 0.02g
	OA1.5 mM	44.51 ± 3.96fg	9.02 ± 0.15h	5.50 ± 0.07cde	2.78 ± 0.04f
ANOVA					
*F* (Storage time)		30.76***	46.9***	14.27***	69.39***
*F* (OA × Storage time)		45.26***	138.4***	55.4***	304.95***

*Note:* Different letters in the same column indicates statistical differences at *p* ≤ 0.05.

Abbreviations: ns, not significant; OA, oxalic acid.

*** *p* ≤ 0.001.

**TABLE 3:** Continuation of Table 2 (mg kg^−1^).

Storage time		Ferulic	Protocatechuic	Vanillic	Caffeic
Harvest		5.15 ± 0.08a	10.62 ± 0.15a	8.05 ± 0.23a	3.73 ± 0.10a
Day 15		4.75 ± 0.07b	7.12 ± 0.47b	6.44 ± 0.12b	3.00 ± 0.06b
Day 30		4.61 ± 0.06b	5.52 ± 0.27c	5.09 ± 0.29c	2.38 ± 0.13c
Day 45		3.92 ± 0.09c	5.05 ± 0.21c	3.98 ± 0.23d	1.86 ± 0.11d
Oxalic acid and storage time interaction					
Harvest		5.15 ± 0.08a	10.62 ± 0.15a	8.05 ± 0.23a	3.73 ± 0.10a
Day 15	Control	4.49 ± 0.07e	5.73 ± 0.12efg	6.09 ± 0.13cd	2.82 ± 0.07cd
	OA 0.5 mM	4.70 ± 0.05cd	6.18 ± 0.13de	6.38 ± 0.15c	3.00 ± 0.08c
	OA 1.0 mM	4.90 ± 0.06b	7.76 ± 0.12c	6.36 ± 0.12c	2.98 ± 0.03c
	OA1.5 mM	4.93 ± 0.03b	8.80 ± 0.17b	6.92 ± 0.11b	3.22 ± 0.06b
Day 30	Control	4.42 ± 0.03ef	4.78 ± 0.13ıj	3.84 ± 0.11hı	1.79 ± 0.05hı
	OA 0.5 mM	4.49 ± 0.04e	5.17 ± 0.15hı	5.22 ± 0.15f	2.46 ± 0.08f
	OA 1.0 mM	4.68 ± 0.04d	5.51 ± 0.20fgh	5.52 ± 0.13ef	2.57 ± 0.06ef
	OA1.5 mM	4.85 ± 0.04bc	6.63 ± 0.22d	5.77 ± 0.12de	2.68 ± 0.06de
Day 45	Control	3.63 ± 0.05h	4.53 ± 0.15j	3.23 ± 0.16j	1.51 ± 0.07j
	OA 0.5 mM	3.78 ± 0.04h	4.60 ± 0.28j	3.66 ± 0.10ıj	1.70 ± 0.04ıj
	OA 1.0 mM	3.99 ± 0.04g	5.27 ± 0.08ghı	4.29 ± 0.10gh	1.99 ± 0.04h
	OA1.5 mM	4.28 ± 0.05f	5.80 ± 0.08ef	4.73 ± 0.29g	2.24 ± 0.12g
ANOVA					
*F* (Storage time)		30.09***	23.46***	34.31***	33.14***
*F* (OA × Storage time)		83.48***	124.87***	81.94***	85.82***

*Note:* Different letters in the same column indicates statistical differences at *p* ≤ 0.05.

Abbreviation: OA, oxalic acid.

*** indicates *p* ≤ 0.001.

The stimulation of the antioxidant system: OA provides detoxification of ROS by increasing the activity of antioxidant enzymes such as SOD, CAT, and POX (Zheng et al. 2007). The maintenance of redox homeostasis: OA supports the structural stability of phenolic compounds by maintaining intracellular redox balance (Wu et al. 2011). Promotion of phenolic compound synthesis and accumulation: OA administration can stimulate the synthesis of new phenolic compounds by activating enzymes in the phenylpropanoid pathway, such as PAL. The similar findings regarding the preservation of phenolic compounds by OA have been reported in other studies. Zheng et al. (2012) showed that OA application suppressed PPO activity, activated antioxidant systems, and preserved phenolic contents for longer periods in fruits such as mango and peach. Wu et al. (2011) reported that OA reduced textural deterioration and phenolic loss in plum fruit by inhibiting enzymes such as PG and PE. Ali et al. (2020) and Zheng et al. (2019) showed that OA prevented browning and stabilized phenolic structures in products such as lotus root and bamboo shoot. Martínez‐Espla et al. (2014) reported that OA increased the amount of bioactive compounds and antioxidant capacity when applied both pre and postharvest in high‐value fruits such as sweet cherries. Huang et al. (2013) reported that OA application in banana fruit contributes to the protection of phenolic structures from oxidative stress by increasing SOD and POD activities. Furthermore, the studies such as Selcuk and Erkan (2015) and Bahar and Lichter (2018) suggest that OA can suppress oxidative degradation more effectively when used in conjunction with controlled atmospheric conditions. In this context, it is reported that OA can provide longer‐term protection not only alone but also when applied in combination with low O_2_ and high CO_2_ conditions (Khan et al. 2020).

### 3.7 Correlation Analysis

**FIGURE 2:**
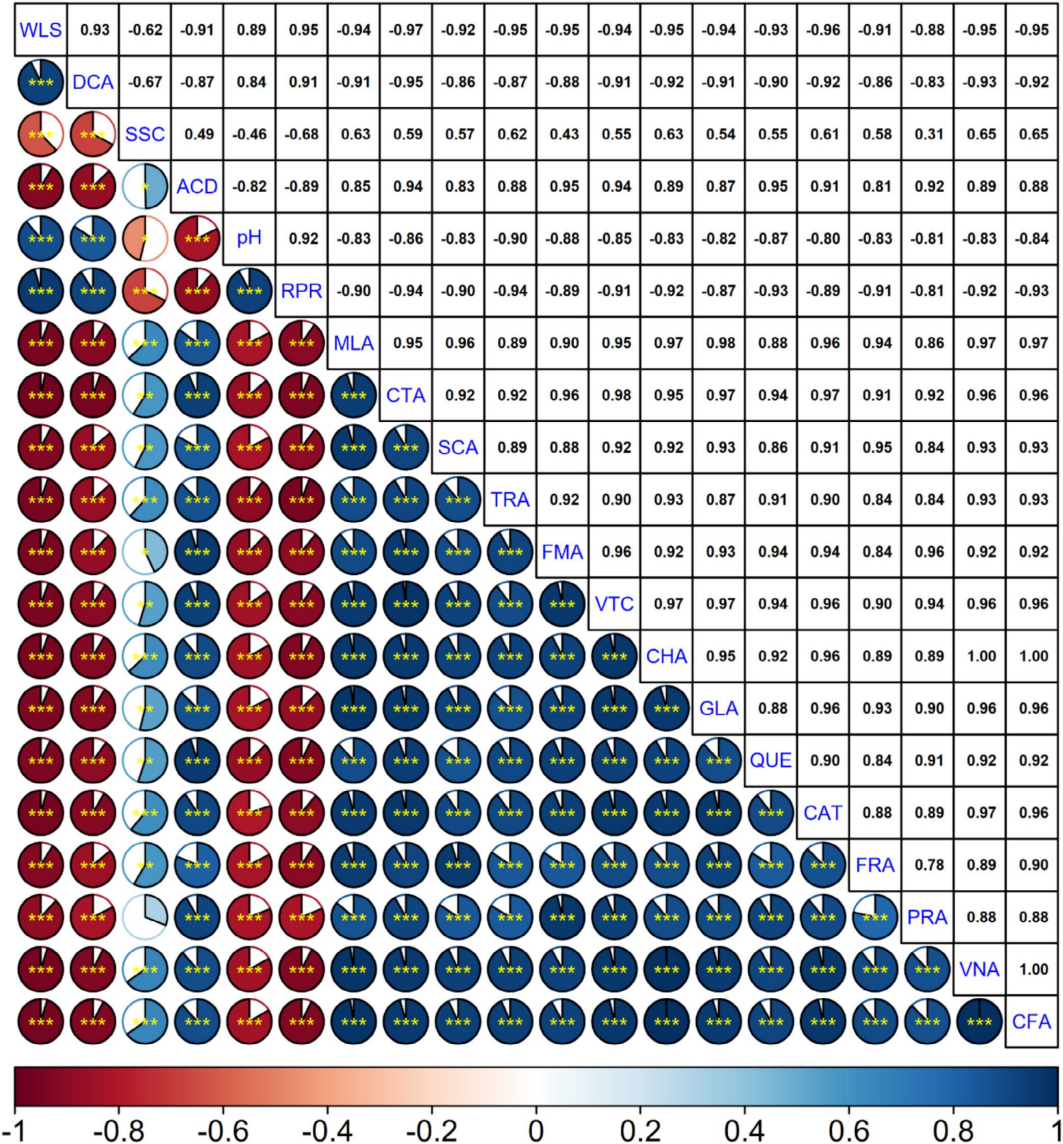
Relationships between quality traits and biochemical contents of medlar fruit during storage period according to correlation analysis. *, **, and *** indicates significance at *p* ≤ 0.05, *p* ≤ 0.01, and *p* ≤ 0.001, respectively. ACD, acidity; CAT, catechin; CFA, caffeic acid; CHA, chlorogenic acid; CTA, citric acid; DCA, decay; GLA, gallic acid; FMA, fumaric acid; FRA, ferulic acid; MLA, malic acid; PRA, protocatechuic acid; RPR, respiration rate; SCA, succinic acid; SSC, soluble solid contents; TRA, tartaric acid; VNA, vanillic acid; VTC, vitamin C; WLS, weight loss.

In this study, the relationships between biochemical compounds and quality changes occurring during the storage process in medlar fruit were evaluated through correlation analysis. The findings revealed significant statistical correlations between both quality parameters and biochemical contents. In particular, the negative correlations were found between parameters indicating quality loss, such as weight loss and decay rate, and some bioactive compounds (e.g., gallic acid, chlorogenic acid, quercetin, and ascorbic acid), indicating that increased physiological deterioration during storage negatively impacts the nutritional value and antioxidant capacity of the fruit. The decrease in phenolic compounds with weight loss suggests that, in addition to water loss, oxidative spoilage processes also play a role in the degradation of these compounds. Similarly, the increase in decay rate is inversely proportional to ascorbic acid content, suggesting that water‐soluble and oxidation‐sensitive compounds, such as vitamin C, rapidly decline during the fruit's spoilage process. The high positive correlations between titratable acidity and organic acids such as malic and citric acid confirm that these acids are the primary determinants of total acidity in the fruit. Furthermore, the positive correlation between succinic acid and acidity demonstrates the contribution of this compound to acidic composition. These findings explain the dynamics of chemical components that directly affect the flavor profile of the fruit during storage. The high positive correlations observed among phenolic compounds (e.g., relationships between chlorogenic acid and protocatechuic and ferulic acid) indicate that these compounds undergo synchronous evolution due to common biosynthetic or degradation pathways. The concomitant decrease in phenolics, particularly catechin, gallic acid, and protocatechuic acid, suggests that these compounds share similar sensitivities to oxidative stress. Positive correlations between respiration rate and weight loss and decay rate suggest that increased metabolic activity accelerates fruit senescence and quality loss. This suggests that optimizing storage conditions to slow respiration rates may be decisive in maintaining fruit quality. In conclusion, the correlation analysis conducted in this study demonstrates that both the quality and nutritional components of medlar fruit change simultaneously and interdependently during storage. The findings provide fundamental data for developing strategies to preserve quality and functional properties, particularly during the postharvest period. In this context, it is recommended that biochemical indicators such as phenolic compounds and organic acids be considered as important quality criteria for storage stability (Figure 2).

### 3.8 Principal Component and Heatmap Analysis

**FIGURE 3:**
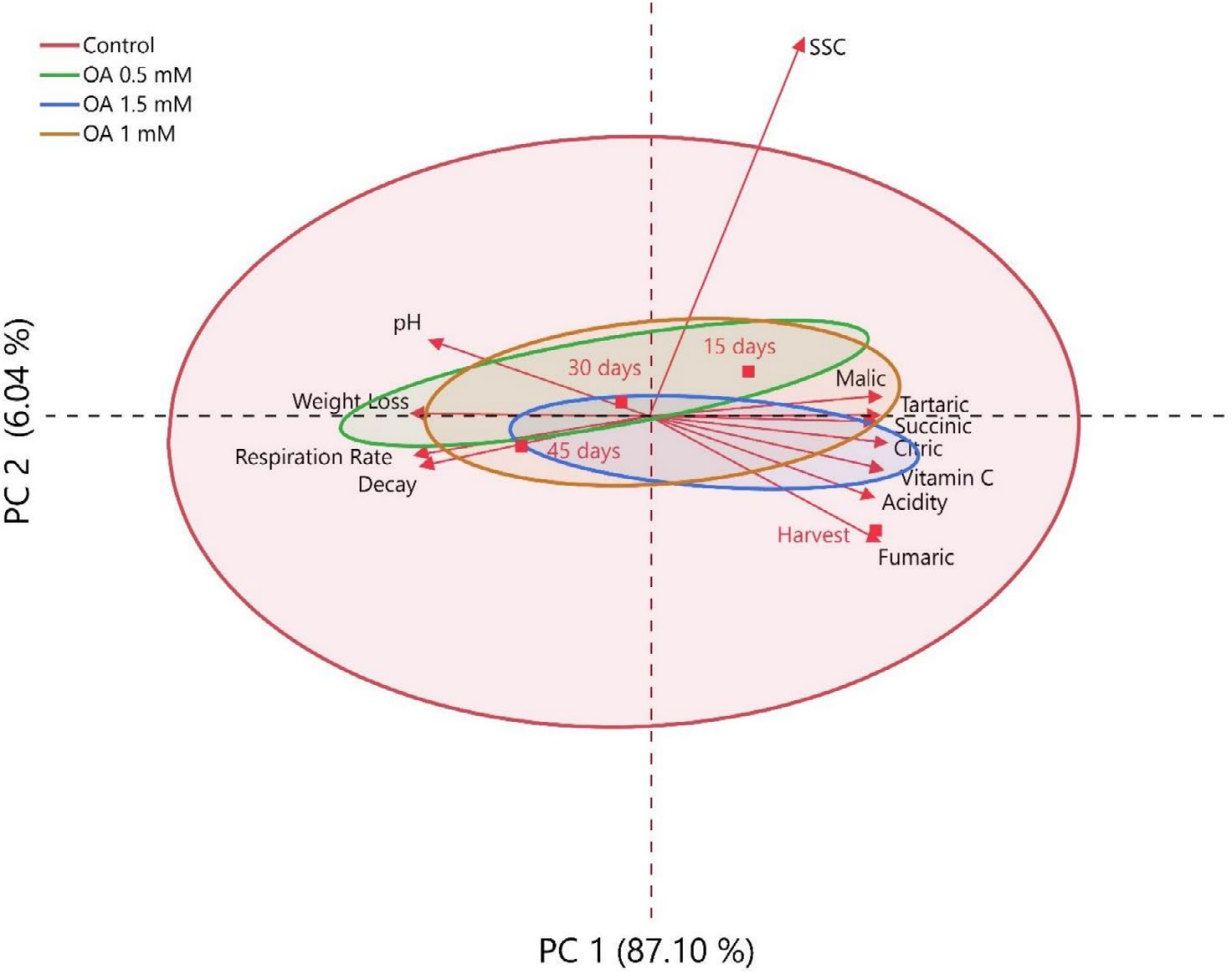
Changes in quality properties and organic acid contents of medlar fruit during storage according to PC (principal component) analysis.

**FIGURE 4:**
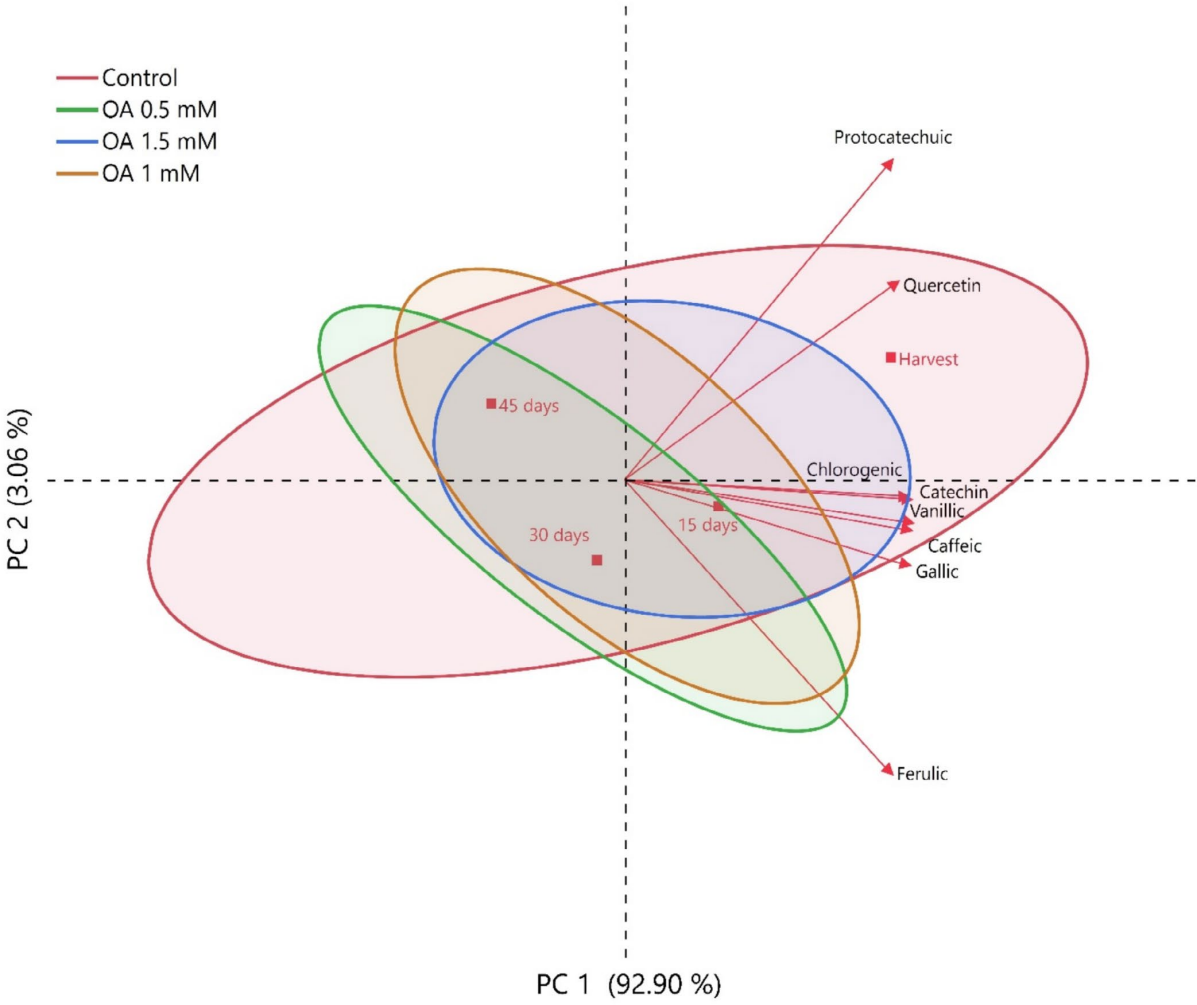
Changes in phenolic compounds of medlar fruit during storage according to PC (principal component) analysis.

**FIGURE 5:**
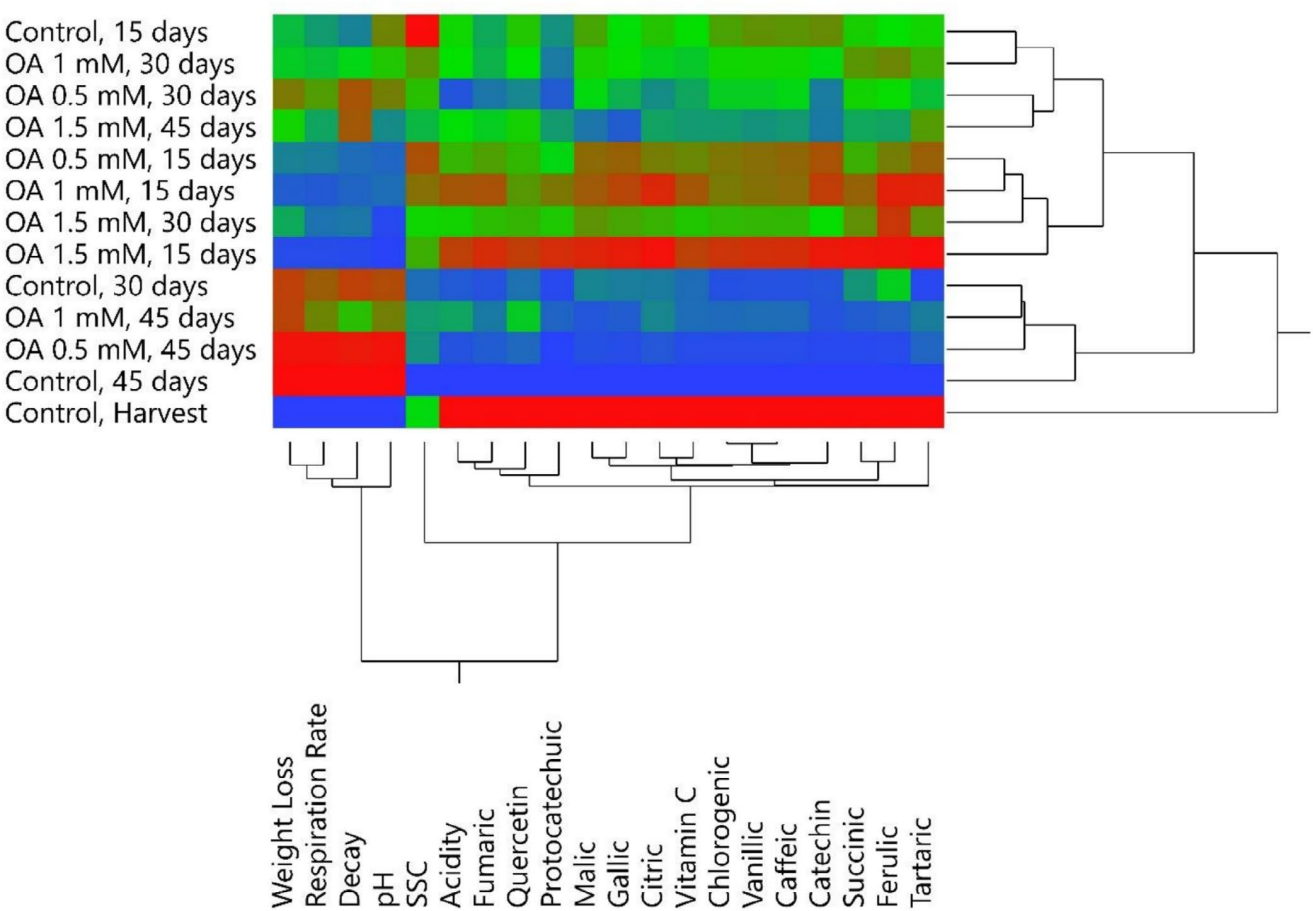
Changes in physicochemical properties of medlar fruit during storage according to Heatmap analysis. The blue to red color scale shows the minimum to maximum values for each properties.

In this study, the relationships between quality losses occurring during storage in medlar fruit and organic acids, phenolic compounds, and other biochemical properties were investigated in detail by multivariate analysis methods, Principal Component Analysis (PCA) and heatmap analysis (Figures 3, 4, and 5). The results revealed that the storage significantly affects both the physicochemical structure and functional component content of the fruit. According to PCA results, during early storage, fruits exhibited high positive correlations with parameters such as malic acid, citric acid, and overall acidity. Although PC1 explained 92.9% of the total variance, PC2 (3.06%) was included to visualize minor quality variations and treatment clustering, which helped distinguish subtle differences between OA doses.

These components play a key role in maintaining the flavor profile and metabolic balance of fresh fruit. However, as storage progressed, samples were associated with parameters that reduced quality, such as weight loss, decay, and respiration rate, indicating that physiological aging and deterioration processes in the fruit accelerated. This finding supports that fruit metabolism negatively affects quality parameters with increasing respiration rate during storage. In the analysis in which phenolic compounds were evaluated by PCA, it was observed that antioxidant compounds such as gallic acid, chlorogenic acid, catechin, and quercetin were found intensely in fresh samples, but there was a significant decrease in the concentrations of these compounds as the storage period progressed. The cumulative contribution rate of PC1 + PC2 was 95.96%, indicating a reliable dimensional representation exceeding the 80% threshold commonly accepted for PCA.

This suggests that phenolic compounds are degraded by oxidative enzymes (especially polyphenol oxidase) and their susceptibility to environmental stressors. Furthermore, the clustering of these compounds and their similar degradation trends suggest common biosynthetic or degradation pathways. These findings clearly demonstrate that the antioxidant capacity of the fruit weakens and functional quality decreases during storage.

Heatmap analysis visually revealed trends in quality parameters and biochemical contents throughout storage. It was determined that ascorbic acid and phenolic compounds, particularly those found at high levels in fresh fruit, decreased significantly between the 30th and 45th days of storage, while weight loss, decay, and respiration rates increased significantly. Additionally, the heatmap grouped the parameters into clusters, revealing components with similar behavior, indicating that phenolic and organic acids decreased together, and degradation indicators increased together. All these analyses demonstrate that medlar fruit undergoes rapid physical and chemical changes during postharvest storage, significantly impacting quality. Given that losses in functional components, in particular, reduce antioxidant potential, which is important for consumer health, it's essential to store this fruit under cold chain conditions and in suitable atmospheres.

## 4 Conclusion

This study demonstrated that OA applications are an effective method for slowing down postharvest quality losses and preserving biochemical components of medlar ( *M. germanica* L.) fruit. Physical deterioration and chemical changes observed during storage were significantly limited by OA applications. In particular, the 1.5 mM OA dose was the most effective concentration in preserving fruit texture and extending shelf life. The correlation analyses showed negative correlations between quality loss indicators and some bioactive compounds and positive correlations between organic acids and acidity. PCA and heatmap analyses confirmed that OA application protected fruit components in early storage and delayed quality deterioration in later storage. Phenolic and organic acids, in particular, were found to be more stable under OA and more resistant to oxidative and enzymatic degradation. OA applications increase both the physical and biochemical stability of medlar fruit, delay quality loss, and preserve its functional components. In conclusion, OA applications significantly enhanced the postharvest storability and biochemical stability of medlar fruit. These findings highlight the potential of OA as an environmentally friendly alternative to synthetic preservatives. Future studies should focus on combining OA with modified atmosphere packaging (MAP) or edible coatings to optimize long‐term storage efficiency and explore molecular mechanisms of OA‐induced stress tolerance.

## Author Contributions

**Ayşen Melda Çolak, Özlem Gündoğdu, Erdal Orman:** conceptualization, methodology, software, formal analysis, investigation, visualization, data curation, and writing‐original draft preparation; **Erdal Aglar** and **Nevzat Sezgin:** methodology, validation, resources, software, formal analysis, investigation, visualization; **Muttalip Gundogdu:** conceptualization, methodology, software, formal analysis.

## Conflicts of Interest

The authors declare no conflicts of interest.

## Supporting information


**Table S1:** fsn371362‐sup‐0001‐TableS1.docx.

## Data Availability

All data generated or analyzed during this study are included in this published article.
