# Supplementary material for: Impact of Postharvest Oxalic Acid Treatments on Quality Properties and Biochemical Composition of Medlar (Mespilus germanica L.) Fruit
Source: Food Sci Nutr. 2026 Apr 15;14(4):e71362. doi: 10.1002/fsn3.71362 (PMC13080689; doi:10.1002/fsn3.71362)
Supplement: Supplementary file 1 — Table S1: fsn371362‐sup‐0001‐TableS1.docx. [file FSN3-14-e71362-s001.docx]

**Supplementary Table 1**. Impact of postharvest oxalic acid application on quality traits of medlar fruits

| Storage time | | Weight Loss (%) | Decay (%) | SSC (%) | Acidity (%) | pH | Res. Rate  (nmol CO_2_ kg^-1^ h^-1^) |
| --- | --- | --- | --- | --- | --- | --- | --- |
| Harvest | | 0.00 ± 0.00c | 0.00 ± 0.00c | 16.68 ± 0.46abc | 1.09 ± 0.04a | 3.70 ± 0.14c | 5.17 ± 0.13c |
| Day 15 | | 1.68 ± 0.32c | 1.38 ± 0.22c | 17.82 ± 0.29a | 0.90 ± 0.02b | 3.98 ± 0.10bc | 6.00 ± 0.15c |
| Day 30 | | 3.78 ± 0.40b | 3.83 ± 0.58b | 16.67 ± 0.26b | 0.76 ± 0.03c | 4.21 ± 0.10ab | 7.09 ± 0.29b |
| Day 45 | | 5.21 ± 0.47a | 5.44 ± 0.50a | 15.78 ± 0.29c | 0.73 ± 0.03c | 4.44 ± 0.11a | 8.14 ± 0.44a |
| Oxalic acid and storage time interaction | |  |  |  |  |  |  |
| Harvest | | 0.00 ± 0.00ı | 0.00 ± 0.00g | 16.68 ± 0.46c-f | 1.09 ± 0.04a | 3.70 ± 0.14f | 5.17 ± 0.13ı |
| Day 15 | Control | 2.80 ± 0.29def | 2.02 ± 0.48de | 18.89 ± 0.67a | 0.83 ± 0.02d | 4.36 ± 0.15bc | 6.46 ± 0.28ef |
|  | OA 0.5 mM | 2.04 ± 0.27fg | 1.55 ± 0.06ef | 17.77 ± 0.21b | 0.86 ± 0.02cd | 3.91 ± 0.10ef | 6.21 ± 0.11efg |
|  | OA 1.0 mM | 1.25 ± 0.17gh | 1.40 ± 0.13ef | 17.54 ± 0.34bc | 0.94 ± 0.02bc | 3.93 ± 0.06ef | 5.80 ± 0.14gh |
|  | OA1.5 mM | 0.63 ± 0.12hı | 0.53 ± 0.07fg | 17.11 ± 0.13bcd | 0.96 ± 0.03b | 3.73 ± 0.05f | 5.52 ± 0.15hı |
| Day 30 | Control | 5.08 ± 0.28bc | 5.32 ± 0.68b | 15.58 ± 0.21g | 0.70 ± 0.01ef | 4.47 ± 0.04b | 8.06 ± 0.19c |
|  | OA 0.5 mM | 4.46 ± 0.18c | 5.08 ± 0.03b | 17.00 ± 0.22b-e | 0.69 ± 0.09ef | 4.37 ± 0.04bc | 7.52 ± 0.14d |
|  | OA 1.0 mM | 2.99 ± 0.26de | 3.11 ± 0.74cd | 17.28 ± 0.19bc | 0.82 ± 0.01d | 4.23 ± 0.06cd | 6.71 ± 0.17e |
|  | OA1.5 mM | 2.57 ± 0.35ef | 1.80 ± 0.26e | 16.84 ± 0.08c-f | 0.83 ± 0.02d | 3.76 ± 0.06f | 6.08 ± 0.09fg |
|  | Control | 6.74 ± 0.08a | 7.20 ± 0.23a | 14.51 ± 0.26h | 0.63 ± 0.01f | 4.79 ± 0.07a | 9.56 ± 0.13a |
|  | OA 0.5 mM | 5.61 ± 0.37b | 5.82 ± 0.36b | 16.06 ± 0.08fg | 0.68 ± 0.02ef | 4.59 ± 0.03ab | 8.78 ± 0.11b |
| Day 45 | OA 1.0 mM | 5.06 ± 0.36bc | 3.73 ± 0.65c | 16.15 ± 0.09efg | 0.77 ± 0.00de | 4.36 ± 0.04bc | 7.71 ± 0.26cd |
|  | OA1.5 mM | 3.45 ± 0.52d | 5.02 ± 0.23b | 16.39 ± 0.21d-g | 0.81 ± 0.02d | 4.01 ± 0.03de | 6.52 ± 0.15ef |
| ANOVA |  |  |  |  |  |  |  |
| F (Storage time) | | 19.95*** | 18.51*** | 9.17*** | 16.86*** | 5.25** | 10.95*** |
| F (OA x Storage time) | | 51.86*** | 33.32*** | 13.78*** | 15.86*** | 20.3*** | 63.22*** |

Different letters in the same column indicates statistical differences at *p*≤0.05. *** indicates *p*≤0.001. OA: Oxalic acid.
